# The cost of systemic corticosteroid-induced morbidity in severe asthma: a health economic analysis

**DOI:** 10.1186/s12931-017-0614-x

**Published:** 2017-06-26

**Authors:** L. E. Barry, J. Sweeney, C. O’Neill, D. Price, L. G. Heaney

**Affiliations:** 1 National University of Ireland, Galway, Ireland; 2 Centre for Infection and Immunity, Queen’s University of Belfast, Belfast, Northern Ireland UK; 3 Centre for Public Health, Queen’s University Belfast, Northern Ireland, Belfast UK; 4 Academic Primary Care, University of Aberdeen, Scotland, UK

**Keywords:** Systemic Corticosteroids, Asthma, Health Economics, Comorbidity

## Abstract

**Background:**

Treatment of severe asthma may include high dose systemic-steroid therapy which is associated with substantial additional morbidity. This study estimates the additional healthcare costs associated with steroid-induced morbidity by comparing three patients groups: those with severe asthma, moderate asthma and no asthma.

**Methods:**

Patients with severe asthma (*n* = 808, GINA step 5 treatment) were matched by age and gender with patients with mild/moderate asthma (*n* = 3,975, GINA step 2 and 3 treatment) and a non-asthma control cohort (with a diagnosis of rhinitis; *n* = 2,412) from the Optimum Patient Care Research Database (OPCRD), a nationally representative primary care database. Prescribed drugs and publicly funded healthcare activity were monetised and annual costs per patient estimated. Regression analyses were used to estimate the additional healthcare cost associated with steroid-induced morbidity.

**Results:**

Average healthcare costs per person per year range from £2603 - £4533 for the severe asthma cohort, to £978 - £2072 for the mild/moderate asthma cohort, to £560 - £1324 for the non-asthma control cohort, depending on the costing scenario. Differences in induced morbidity costs were evident between patients with asthma differentiated by steroid exposure. In relation to prescription drugs used to treat steroid-induced co-morbidities, females with severe asthma and high steroid exposure cost approximately £789 more per year than a corresponding female with no asthma, while males cost approximately £744 more than their counterparts with no asthma. Estimates were extrapolated to all healthcare costs.

**Conclusions:**

This study provides the first robust estimates of the additional cost of healthcare related to steroid-induced morbidity relative to patients with no steroid exposure. The study will help inform use of steroid-sparing strategies in this patient group.

**Electronic supplementary material:**

The online version of this article (doi:10.1186/s12931-017-0614-x) contains supplementary material, which is available to authorized users.

## Background

Asthma is estimated to affect up to 334 million people worldwide [1] and presents a substantive burden in terms of morbidity, mortality and economic costs. Estimates of the economic burden vary but in the United States (US) and Europe the annual figure is measured in the billions, both in US Dollars and Euros [2–4].

Patients with Severe Asthma (SA) contribute disproportionately to the overall burden [5, 6]. They have higher levels of healthcare utilization including unscheduled primary care visits and hospital admissions, along with greater drug therapy consumption and diagnostic procedures relative to those with mild asthma and non-asthmatics [7]. While previous research has focused on ascertaining the burden of disease associated with asthma and variations across patients with differing disease severity, an area of increased interest concerns the cost specifically associated with corticosteroid-induced morbidity.

Prolonged exposure to corticosteroids among asthma patients particularly severe asthmatics, has been implicated in a range of morbidities including hypertension, diabetes, arthritis and osteopenia [8]. A higher prevalence of these and other conditions has been demonstrated among those with higher exposure to corticosteroids [9]. It has been shown also that among those with SA, drug costs are the main driver of total healthcare costs and costs unrelated to asthma were higher for patients on maintenance oral corticosteroids (OCS) [7].

Others have examined differences in the cost of managing specific comorbid conditions between groups with high (>12 mg/day), moderate (6-12 mg/day) and low (≤6 mg/day) corticosteroid exposure, (although a non-exposure control group was not used in this analysis) [10]. This work has shown that the costs associated with managing a list of specific conditions related to systemic corticosteroids is USD $1194 and $5479 higher among medium and high exposed groups relative to a low exposure group respectively [10].

In this paper we examine differences in healthcare costs related to corticosteroid exposure between patient groups with and without asthma (diagnosed with rhinitis) as well as differentiated by asthma severity within the asthma group. We use nationally representative data from the UK and focus attention on the relationship between morbidity and corticosteroid exposure to better understand the impact of systemic corticosteroid exposure on the cost of managing comorbid conditions.

## Methods

### Cohort definition

Patients with SA (*n* = 808) requiring regular OCS (defined as Global Initiative for Asthma (treatment at GINA) step 5 treatment [10, 11] and ≥4 prescriptions for OCS in each of two consecutive study years) were compared with patients with mild/moderate asthma (*n* = 3975) and non-asthmatic controls (*n* = 2412). Data were obtained from the Optimum Patient Care Research Database (OPCRD) a large nationally representative primary care practice database [8]. Subjects were required to be over 12 years of age and to have at least 2 years of continuous medical records, so that 24 months of continuous primary care data were available for analysis. Two control cohorts were individually matched to the SA cohort for age, gender and year of birth (due to differing data extraction dates – data were extracted between 2008 and 2013), one with mild/moderate asthma (asthma diagnosis, treatment at GINA step 2/3 [11]), the other consisting of non-asthmatic controls.

Patients were included in the non-asthmatic control group if they had a rhinitis diagnosis with no asthma diagnosis/asthma drug prescription and no exposure to OCS as evidenced by the patterns of service use in OPCRD. To avoid any risk of non-asthma related exposure to corticosteroids, subjects with the following conditions for which systemic corticosteroids may be prescribed were removed from the data: Crohn’s disease, ulcerative colitis, autoimmune hepatitis, rheumatoid arthritis, polymyalgia rheumatic, chronic obstructive pulmonary disease, bronchiectasis, cystic fibrosis, interstitial lung disease, tuberculosis. Thus the mild/moderate asthma group had some OCS exposure and had inhaled corticosteroid (ICS) exposure. As such the change in cohort from non-asthma to mild/moderate asthma to severe asthma represents a change in corticosteroid exposure from none to low to high exposure. The data included details of all publicly funded healthcare consultations, including primary and hospital care as well as details of prescribed therapies/drugs.

### Monetising data – healthcare activity

A list of all interactions with health service personnel (healthcare activities), detailed in Read codes (RC) [12], were extracted from the database. RC’s are used by clinicians to report details on interactions with patients including procedures undertaken as well as referrals made; codes are arranged in a hierarchical fashion, all codes beginning with three for example denoting Diagnostic Procedures (see Additional file 1: Table S9); the RC 3361.00 denoting, more specifically, ‘Allergy Testing Done’. At lower levels of the hierarchy, RC’s may be elaborated with additional detail entered in records as free text.

As such, the monetisation required an approach tailored very specifically to this dataset. Full details of this are available in the supplement but in summary, monetisation of interactions involved a series of steps; first, an algorithm was developed and used to assign a consultation type to each RC. For example, ‘asthma assessment’, might initially be assigned a General Practice (GP) consultation based on where the procedure was likely to be undertaken – a GP surgery. Next, algorithm based assignments were independently reviewed by two researchers (JS and CON) and based on an examination of the RC, assignments were confirmed or amended. Where uncertainty remained as to the appropriate assignment, for example where a practice nurse or a GP could have performed a particular activity, high-low cost scenarios were developed based on alternative possibilities and used in subsequent sensitivity analyses. Activity which was documented as occurring outside the GP was also costed, for example outpatient visits, X-Rays or GP administration.

Confirmatory analysis of healthcare activity costing was undertaken on a random sample of 22 study subjects across cohorts in which all levels of the hierarchy were used to infer an assignable unit cost. Cost estimates based on this were compared with those based on the algorithm using a correlation coefficient.

Activity was monetised by reference to Personal Social Services Research Unit (PSSRU) 2013 unit cost estimates [13]. The PSSRU presents median and inter-quartile range (IQR) estimates for consultation types. This range was used as part of sensitivity analyses undertaken. Where IQR’s were not provided, a range of costs were calculated based on the value reported in the PSSRU and using a standard gamma distribution (θ ∼ gamma (α,β)), commonly used for cost data [14], with mean and variance as parameters of 10,000 simulated observations for sensitivity analyses.

This resulted in six potential scenarios for the monetisation of each healthcare activity; the median, the upper and lower quartile PSSRU (or gamma distribution) estimates for the high and low cost scenarios where uncertainty existed as to the appropriate activity to relate to a given RC.

### Monetising data – prescription drugs

British National Formulary (BNF) codes for prescribed medicines were recorded in OPCRD. The code identified a group of drugs rather than a specific brand and dose. Unit costs for the group at the most disaggregated level identified were taken from the Northern Ireland Prescription Cost Analysis (PCA) [14]; the mean and median of the unit costs in the relevant section of PCA were then applied to the code resulting in two prescription cost scenarios. Where there was evident uncertainty as to the unit cost applied (based on the coefficient of variation in unit costs for the group of drugs) or where the drug was frequently prescribed and misallocation could have a material effect on results (>200 cases were observed to be prescribed), a manual exercise was undertaken linking individual drugs to the PCA.

A confirmatory analysis was subsequently undertaken in which 50 patients per cohort had drug costs estimated as described above. All drug costs for these patients were also manually costed based on the string descriptors for their prescriptions and results compared. One patient exhibited very high prescription costs and upon further investigation it was found that they did not have any prescription quantities present and was therefore entirely imputed. This individual was deemed an outlier and excluded from further analysis.

Further details of the costing of the data, outlier investigation and validation analyses undertaken are available in the Additional file 1.

### Data analysis

Differences in cost between groups differentiated by corticosteroid exposure may not accurately describe corticosteroid-induced morbidity costs. Thus, as different patterns of morbidity unrelated to corticosteroid exposure may exist between groups it is important to disentangle morbidity related to corticosteroids and morbidity that is unrelated. To do this we used a two stage regression analysis analogous to that of McGregor et al. [15] and Doherty et al. [16].

In the first stage the relationship between corticosteroid exposure and morbidity was determined using a zero-inflated Poisson model to account for individuals without any comorbidity (22% of the sample). Here the number of comorbidities (as identified in Table 1 and [17]) experienced by an individual was regressed on cohort, gender, age group and region, with just cohort membership in the zero-inflated model to predict the likelihood of having one or more comorbidities. Age group was partitioned using an approximate quartile split of the sample (<46 years, 45–60, 61–70, >70). The model allowed us to predict the number of additional morbidities individuals would expect to experience as their corticosteroid exposure (as defined by cohort membership) increased. The residual of this regression – the number of morbidities unexplained by differences in corticosteroid exposure – provides an estimate of morbidity unrelated to corticosteroid exposure, what might be considered as background morbidity.

**Table 1:** Demographic, comorbidity and corticosteroid exposure details across cohorts

Demographics	All (*n* = 7195)	Non-asthma controls (*n* = 2412)	Mild/Moderate asthma (*n* = 3975)	Severe asthma (*n* = 808)	p-value†
Female, n (%)	4503 (63)	1481 (61)	2515 (63)	507 (63)	0.33
Age (years)^a^	58 ± 17	58 ± 17	58 ± 16	59 ± 17	0.65
Geographical region, n (%)					
London	597 (8)	198 (8)	344 (9)	55 (7)	0.22
South of England	903 (13)	333 (14)	477 (12)	93 (12)	0.07
East of England	1064 (15)	333 (14)	616 (16)	115 (14)	0.16
Midlands	2146 (30)	685 (28)	1213 (31)	248 (31)	0.17
North of England	1648 (23)	581 (24)	874 (22)	193 (24)	0.12
Scotland/NI/Wales/unknown	837 (12)	282 (12)	451 (11)	104 (13)	0.47
Number of corticosteroid-related comorbidities, n (%)					
Type II diabetes	512 (7)	149 (6)	281 (7)	82 (10)	0.0007
Obesity (Body Mass Index >30)	2285 (32)	561 (23)	1385 (35)	339 (42)	<0.0001
Osteopenia	204 (3)	41 (2)	85 (2)	78 (10)	<0.0001
Osteoporosis	362 (5)	74 (3)	162 (4)	126 (16)	<0.0001
Fracture	263 (4)	88 (4)	134 (3)	41 (5)	0.06
Dyspeptic disorders	3342 (46)	851 (35)	1874 (47)	617 (76)	<0.0001
Glaucoma	236 (3)	67 (3)	137 (3)	32 (4)	0.10
Cataract	370 (5)	105 (4)	195 (5)	70 (9)	<0.0001
Cardiovascular disease	522 (7)	168 (7)	277 (7)	77 (10)	0.03
Hypertension	2017 (28)	596 (25)	1145 (29)	276 (34)	<0.0001
Psychiatric conditions	2155 (30)	607 (25)	1238 (31)	310 (38)	<0.0001
Hypercholesterolemia	943 (13)	258 (10)	561 (14)	124 (15)	<0.0001
Sleep disorder	172 (2)	40 (2)	99 (3)	33 (4)	0.0003
Chronic kidney disease	619 (9)	167 (7)	342 (9)	110 (14)	<0.0001
Inhaled Corticosteroid Dose in Beclomethasone Diproprionate (BDP) equivalents^a^	----	----	499 ± 323 (*n* = 3898)	1411 ± 846 (*n* = 738)	<0.0001
OCS Prescriptions per year^a^	----	----	1.2 ± 1.8 (*n* = 995)	11 ± 9 (*n* = 808)	<0.0001

Mean values of inhaled and oral corticosteroids relates only to those with corticosteroid exposure

†P-value’s relate to an analysis of variance between samples (F-test) where three or more samples are being tested, otherwise the equality of means (*t*-test) was used

^a^Mean ± Standard Deviation (SD)

In the second stage a generalised linear model (GLM) with power link function of 0.5 and Poisson distribution demonstrated best fit for this right skewed cost data and was used to examine the relationship between non-asthma drug costs and corticosteroid exposure (as captured by cohort membership). The same covariates from the Poisson model above were used, along with the residual from the first stage regression (morbidity unrelated to corticosteroid exposure) and an interaction term between age group and cohort membership.

This model explores the relationship between non-asthma drug costs and corticosteroid exposure controlling for differences in background morbidity (i.e. morbidity unrelated to corticosteroid exposure). The regression focused on non-asthma drug costs, that is the median overall drug cost (the lower of the two prescription cost scenarios described above) minus the median cost of asthma drugs per patient, as this is a category of cost we can be certain is not directly asthma related and for which we can be as certain as possible we are examining induced as opposed to direct asthma morbidity. By contrast with GP consultations, for example, while a consultation may relate to a diabetes clinic, unless the consultation were actually recorded there is no way of knowing definitively whether asthma was not discussed or advice given as part of the consultation and whether therefore there were not elements of direct as well as induced morbidity.

Differences in cost based on the regression analyses were calculated across a range of variables to highlight differences between specific groups. Sensitivity analyses examined the impact on estimates of high and low cost scenarios arising from uncertainty related to the attribution of activity and to the level of unit cost assigned to that activity. Analyses were performed using Stata 14.

## Results

Demographics for all patients and associated systemic corticosteroid comorbidities have been described before [9] and are presented in Table 1 below differentiated by group.

Confirmatory analyses related to the validation of activity costs are reported in detail in Additional file 1 but in summary resulted in a correlation coefficient between manually costed data and algorithm costed data of greater than 0.97 and as such the costing methodology was deemed acceptable.

**Fig. 1:**
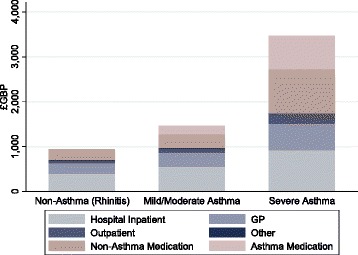
Mean annual healthcare activity and prescription drugs costs. Mean annual healthcare costs by service group across cohorts (For a full list of activities grouped under each service, see Additional file 1: Table S4), along with asthma related and non-asthma related prescription drug costs. Costs are calculated as the average across High-Low cost scenarios; bar height represents the average of this annual cost per patient per group. ‘Other’ includes physiotherapists, psychiatrists, and opticians, among others and accounted for such a small proportion of total healthcare costs that it cannot be seen on the graph

Costs per patient in Table 2 are presented on a per year basis, i.e. the average over the two years. Results from an analysis of variance (F-test) demonstrated statistically significant differences in both sets of costs across cohorts (p < 0.01). Costs increase as corticosteroid exposure increases, with drug costs accounting for a greater proportion of overall costs as this occurs (Fig. 1).

**Table 2:** Mean annual costs per patient for highest and lowest cost scenarios across cohorts

	Non-asthma (*n* = 2411)		Mild/Moderate asthma (*n* = 3975)		Severe asthma (*n* = 808)	
	Low	High	Low	High	Low	High
Clinical activity^a^	£ 350 ± 546	£ 1111 ± 2372	£ 491 ± 630	£ 1579 ± 2902	£ 911 ± 907	£ 2799 ± 3705
Prescription drugs^a^	£ 210 ± 790	£ 212 ± 700	£ 487 ± 957	£ 493 ± 947	£ 1692 ± 2369	£ 1734 ± 2346
Total cost	£ 560	£ 1324	£ 978	£ 2072	£ 2603	£ 4533

Six scenarios were created for clinical costs to capture uncertainty around the likely consultation to be costed (2 scenarios) and median, and IQ ranges (3 scenarios)

^a^Mean ± SD

**Fig. 2:**
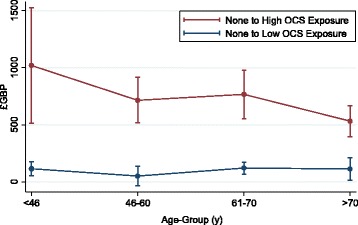
Adjusted differences, between OCS exposure groups, in annual non-asthma prescription costs per patient across age-groups. Difference in annual non-asthma drug cost per patient at each age-group between those with high OCS exposure and those without OCS exposure (*red*) and between those with low OCS exposure and those without OCS exposure (*blue*). Differences in costs per patient are adjusted for confounders (sex, region and background morbidity) and calculated holding confounders at the sample mean. Outer lines represent 95% confidence intervals around these estimates

Results from the first stage regression showed a statistically significant and positive relationship between the level of corticosteroid exposure (evidenced by cohort membership) and the number of comorbidities; further details can be found in the Additional file 1. Second stage regression analysis identified that, when holding confounders at their mean levels (to reduce the impact of extreme values), there is a statistically significant increase in annual non-asthma drugs costs per patient associated with corticosteroid grouping. Fig. 2 presents the adjusted cost differences across age groups, where it can be seen that there is a decreasing pattern in cost differences related to age.

Table 3 presents estimated corticosteroid-induced morbidity non-asthma drug costs differentiated by males and females, as well as males and females combined per year, arising from the regression analyses. Holding other covariates at the sample mean, females with high corticosteroid exposure (severe asthma) would cost approximately £789 more per year on average than a corresponding female with no exposure (non-asthma). The average male from the sample with high exposure would cost approximately £744 more per year than a corresponding male with no exposure.

**Table 3:** Adjusted difference in non-asthma drug costs per patient per year relative to control group

Base: No OCS exposure (Non-asthma control)		Adjusted cost differences	Lower bound CI (95%)	Upper bound CI (95%)
Low OCS exposure (Mild/Moderate asthma)	Female	£ 115^a^	£ 82	£ 148
	Male	£ 106^a^	£ 77	£ 135
	Total	£ 112^a^	£ 80	£ 143
High OCS exposure (Severe asthma)	Female	£ 789^a^	£ 652	£ 927
	Male	£ 744^a^	£ 620	£ 868
	Total	£ 772^a^	£ 641	£ 903

Data are presented for males, females and with gender at the sample mean (total)

^a^Significant at α = 0.01

## Discussion

The data in this manuscript provides estimates of the additional cost of non-asthma related prescribed medicines associated with corticosteroid-induced morbidity. The data demonstrates how much more SA costs relative to those with moderate or no asthma and how much of this is as a result of morbidity induced by corticosteroid exposure. The corticosteroid-induced prescription costs are on average approximately £112 more for those in the moderate exposure group and £772 for those in the high exposure group. If we assume a similar pattern in respect of clinical activity as for prescriptions, then 51% of the difference in clinical activity costs can be attributed to corticosteroid-induced morbidity for the SA group and 39% for the moderate asthma group. The average activity costs (across the 6 scenarios described above) associated with corticosteroid-induced morbidity would therefore be £538 and £112, respectively. The estimated annual cost of corticosteroid-induced morbidity, for prescription drugs and healthcare activity combined, would amount to £1310 (£772 plus £538) for the high exposed group and £224 (£112 plus £112) for the low exposure group.

Differences in morbidity costs, even after controlling for morbidity unrelated to corticosteroids and other confounders (region), are higher among women than men and among younger age groups (although confidence intervals overlap – Fig. 2). Essentially the data demonstrates that differences in cost between those with high exposure to OCS and those with no exposure are lower in older age groups. Although research on this age-related cost differential topic is sparse, this effect has been noted elsewhere [18].

This may have important implications specific to the management of asthma. While differences in cost related to gender are unlikely to materially affect evaluations of corticosteroid-sparing therapies, differences related to age could point to possible differences in the cost-effectiveness of corticosteroid-sparing therapies related to age with greater cost-effectiveness in younger patients. We acknowledge that our analysis is cross sectional by design and longitudinal data on corticosteroid exposure and morbidity would better help to understand the relationship between corticosteroid exposure, cost and age.

Severe asthmatics were defined in this study in part by their consumption of ≥4 prescriptions for OCS. Such patients meet the current United Kingdom National Institute for Clinical and Healthcare Excellence criteria to access both omalizumab and mepolizumab (biologic therapies which have some systemic steroid sparing activity) [19]. As noted, our estimates of the cost of corticosteroid-induced morbidity are therefore potentially pertinent to the estimated cost-effectiveness of such therapies. While estimating the cost-effectiveness of corticosteroid-sparing therapies is beyond the scope of this paper, the inclusion of potential savings related to corticosteroid-induced morbidity estimated in this study would serve to improve the estimated cost-effectiveness of such therapies.

Lefebvre et al’s US based study 2016 [18] found that a high-dose systemic corticosteroid (SCS) (>12 mg/day) group would cost approximately £5,949 more in terms of both drugs and activity, per year than a non-SCS exposure group (data was published in 2013 US Dollars and have been converted into 2013 Great British Pounds (GBP), adjusting for purchasing power parity [20]). These estimates are somewhat higher than those produced here, though it is difficult to make direct comparisons between the two studies given differences in study settings, healthcare systems under which patients healthcare costs are incurred (the US has higher healthcare costs [21]), and different costing methodologies. The data used by Lefebvre et al. are confined to a selected set of US states and to Medicaid recipients within those states, which may also influence estimate of induced morbidity costs. For example, Medicaid recipients may exhibit poorer general health, different patterns of service use and outcomes, both among those with corticosteroid exposure and between those with and without such exposure. Whether the estimates are generalisable is therefore unclear. It is also noted that the authors’ measure differences in resource use relative to a group without corticosteroid exposure which are not matched by age and consequently the mean age of the exposure group (57.6 years) is more than twice the mean age of the group without exposure (27.4 years), which may also make inference from these results less reliable. A strength of our data is that we are able to estimate costs unrelated to asthma among a nationally representative group of patients including a control group, matched by age, gender and year of birth, without corticosteroid exposure.

Our study does have limitations. Firstly, the large dataset required the estimation of costs, described above, which in turn required some assumptions and clinical judgement. However steps were taken to reduce errors in estimation and possible biases that may have arisen in our approach; based on the confirmatory and sensitivity analyses (see supplement), we do not expect that the data would be biased in a systematic way.

Secondly, the first stage regression investigates the effect of corticosteroid exposure on the number of comorbidities in order to identify the impact on comorbidity which is potentially unrelated to corticosteroid exposure (the residual from this equation). Because the number of comorbidities is used, rather than each comorbidity and its specific severity separately, they are given equal weighting. This may lead to increased variance in the costing estimates provided. Weights to account for difference in severity of conditions, such as the Charlson comorbidity index [22] were considered however differences in our data and the data required to calculate the index would require many additional assumptions. This could therefore be less reliable than our current estimates and was not considered a worthwhile exercise in this case.

Thirdly, the estimates in this manuscript focus on non-asthma prescription drug costs where the opportunity to disentangle morbidity and induced morbidity costs is more straightforward. Separating healthcare activity costs due to asthma from healthcare activity due other morbidities is not straightforward. Estimates of increased healthcare activity associated with asthma related comorbidity were attempted in a Canadian study but cost estimates were not provided [23]. While extrapolations to other healthcare activity are offered here these are for illustrative purposes, a prospective and more detailed examination of the impact of corticosteroid exposure on ambulatory and hospital care costs remains a subject for further research.

## Conclusion

This paper estimates the additional cost of non-asthma related prescribed medicines and healthcare activity associated with corticosteroid-induced morbidity in severe asthma. Corticosteroid-induced morbidity costs are higher among women than men and among younger patients suggesting that younger age groups facing a greater economic burden of corticosteroid related comorbidity relative to older age groups. The potential savings associated with corticosteroid-induced morbidity which would be avoided through use of effective corticosteroid-sparing treatments should be incorporated in models which estimate the cost-effectiveness of such therapies. These savings are important in considerations of the relative value for money of such therapies and in decisions around which groups should be afforded access to such therapies.

## Additional file

Additional file 1: The cost of systemic steroid induced morbidity in severe asthma. (DOCX 276 kb)

## Abbreviations

BDP: Beclomethasone Diproprionate
BNF: British National Formulary
GBP: Great British Pounds
GINA: Global Initiative for Asthma
GLM: Generalised linear model
GP: General Practice
ICS: Inhaled corticosteroids
IQR: Inter-quartile range
OCS: Oral corticosteroids
OPCRD: Optimum Patient Care Research Database
PCA: Prescription Cost Analysis
PSSRU: Personal Social Services Research Unit
RC: Read codes
SA: Severe asthma
SCS: Systemic corticosteroid
SD: Standard deviation
US: United States
