# Supplementary material for: The cost of systemic corticosteroid-induced morbidity in severe asthma: a health economic analysis
Source: Respir Res. 2017 Jun 26;18:129. doi: 10.1186/s12931-017-0614-x (PMC5485660; doi:10.1186/s12931-017-0614-x)
Supplement: Additional file 1: — The cost of systemic steroid induced morbidity in severe asthma. (DOCX 276 kb) [file 12931_2017_614_MOESM1_ESM.docx]

**The cost of systemic steroid induced morbidity in severe asthma*: Supplementary Material***

**Therapy/Prescription/Drug Data**

In order to calculate the cost of therapies relating to patient activities, we must know the therapy that is being prescribed, the quantity of the therapy and the cost of this therapy. Costs are taken from the Northern Ireland Prescription Cost Analysis (PCA) (1). This expenditure data is used to provide costs per prescription and costs per unit (number of units of a drug per prescription) for a wide variety of products. These unit costs and prescription costs are then matched to the OPCRD sample data using their BNF codes (see ‘Drug Costing’ below). In estimating use and cost of drugs from the data there were issues in terms of incomplete data and heterogeneity in the units reported by GPs, for example some report grams, others packets, others number of tablets.

**Missing Data**

To address this “missingness” issue utilisation of the same drug by others, within the cohort from which the individual was drawn, was used to predict utilisation. In some circumstances information on utilisation by others within the same cohort may not have been present in the data to predict an individual’s missing utilisation; that is no one else in the cohort had been prescribed the same drug.

**Figure 1:** Schematic diagram to demonstrate the values which were compared (area Q) as part of the validation exercise for the missing value imputation.

Thus among the control (rhinitis) group, a pragmatic approach was taken to expand the sample used to all those on whom data existed, i.e. not exclude those with a diagnosis of asthma or use of steroid, rather than resort to using individuals from the other groups to predict missing values because of the absence of a comparator in the control group. Similarly, on occasion individuals were taken from the expanded rhinitis group to predict the utilization of those in other groups where no other comparator in their own group could be found. The expanded rhinitis group comprised 3396 persons, compared with the 2412 sample used in the final comparative analysis of costs.

A validation exercise was undertaken whereby the mean and standard error for prescribed use was predicted using the expanded and un-expanded sample, for only those values which were missing in both samples (area Q in Figure 1). If no material difference is observed in the mean and dispersion then using the expanded sample for missing value imputation would have the advantage of providing more similar values with which to predict missing values (rather than using moderate or severe asthmatics to predict prescribed use). The results are reported in Table 1 where Q1 refers to estimates based on the smaller sample and Q2 on the larger expanded sample. As can be seen there are no material differences between the two methods.

**Table 1**: Mean and standard error comparison between imputation approaches.

|  |  | **Rhinitis** | **Mild Asthma** | **Severe Asthma** | **Total** |
| --- | --- | --- | --- | --- | --- |
| **Mean** | **Q1** | 62.96 | 60.04 | 51.38 | 55.89 |
|  | **Q2** | 64.01 | 59.87 | 51.57 | 56.00 |
| **Standard Error** | **Q1** | 207.00 | 133.98 | 102.38 | 159.47 |
|  | **Q2** | 212.04 | 133.71 | 103.91 | 160.28 |

In respect of quantities being imputed, as noted, there existed heterogeneity in terms of the way in which these were reported in respect of products. With the additional control patients the number of missing values was 385482 of a total of 790445 observations on prescribed drug use (It is worthwhile to note that the proportion of missing values, were the additional control patients to be removed, stays approximately the same throughout each of the following steps). The process of imputation proceeded as follows:

Where there existed observations within the cohort upon which to base imputations the modal unit for the drug concerned was used to impute that for the missing observation. Following this step the number of missing values was reduced from 385482 to 21740.

1. Where no comparators existed within the cohort to which the member belonged, the mode for the entire sample was used, i.e. using comparators from other cohorts as well (reduced from 21740 to 7644).
2. Where no comparator existed for the specific drug prescribed in the entire sample, the modal unit for that form of drug (e.g. tablets, injections, etc.) was imputed from the cohort to which the individual belonged (7644 to 667).
3. Where no comparator existed for the form within each cohort, the modal unit for that form of drug was imputed across all cohorts (667 to 469).
4. For a very small number of cases, where no comparator existed for drug form across all cohorts, the modal unit for all drugs across all cohorts was imputed (469 to 0).

**Scale Heterogeneity**

In relation to the heterogeneity in the scale of the quantities of drugs reported by GPs, the drugs were grouped by their form (e.g. tablets, injections, etc.) and cut-off points were identified to separate quantities by their scale. The PCA presents data as cost per unit and cost per packet and, as such, the cut-off points were chosen to isolate those drugs which are multiplied by the cost per packet or cost per unit. Different cut-off points were applied to different groups of forms, for example the same cut-off was used for both tablets and capsules and another cut-off was used for creams, pastes and gels (full details in appendix 2). Any quantities which were less than or equal to the cut off were multiplied by the cost per packet and anything over were multiplied by the cost per unit.

The data was then inspected to identify very small and very large costs, which were a result of scale heterogeneity persisting. In these instances, it was found that a third scale existed for these products, for example a product may have been reported as packets (1,2,3) or as tablets (12,24,48) or also in milligrams (500,1000,2000). These very high values were therefore rescaled according to the base value of the third group (e.g. 500/500, 1000/500, 2000/500) and then treated as packets.

In the case of very small costs it was found that these related to instances involving less than £1. Analysis was run with these very small values or with rounding these values less than £1 to the value of the smallest unit of that drug for which a cost could be linked from the PCA. It was found that the rounding approach had no material impact on results in the sample and therefore the simple rounding approach was adopted based on its simplicity and ease of application.

**Monetising Prescription Data**

When multiplying the quantity of the drug prescribed by the cost of that drug, issues were encountered as to the appropriate cost to apply. An initial merge between the description of the drug (i.e. the product name) in the dataset and the description in the PCA was deemed too unpredictable due to slight differences in of the string variable (product name) between datasets. As such the following process was developed:

1. The list of all drugs was extracted from the data, which resulted in approximately 5000 drugs to be costed, this included individual product brands along with varying versions of these products, for example a certain brand of dressing may have had a number of similar dressings of varying dimensions which would have to be costed individually.
2. Using the PCA dataset, a weighted average cost per unit and cost per prescription across British National Formulary (BNF) codes was created (2). This was weighted by the quantity of units consumed (i.e. the cost is weighted towards the most commonly used drugs in Northern Ireland).
3. A merge was performed to match the cost of each drug (weighted average across BNF codes) in the PCA data with the list of drugs in the OPCRD sample using BNF codes (common to both datasets). A number of iterations of this process were undertaken to match as many costs as possible to drugs. For example where no match occurred for a specific BNF code at the sub-paragraph level (e.g. 3010101) then a second iteration attempt to match BNF codes at the paragraph level (e.g. 30101) or at the sub-chapter level (e.g. 301).


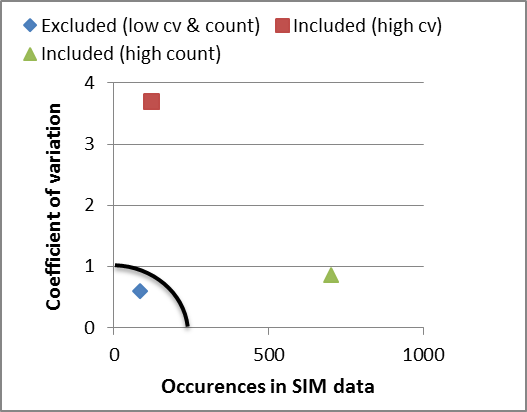


**Figure 2**: Schematic diagram of inclusion criteria for manually costing of prescriptions

1. A manual costing process was then used to account for uncertainty in the process above, so where:
   1. BNF codes varied for a single drug (most likely due to different versions of the BNF used in the longitudinal data)
   2. The coefficient of variation (standard deviation/mean) from the weighted average cost across BNF codes was greater than 1 and/or the drug occurred more than 200 times in the data (see Fig2)
   3. No match occurred in the initial iterations
2. Finally a list of drugs was leftover to be manually costed. This involved performing a search for drugs in the PCA dataset or for a proxy (e.g. a different brand of the same product) and manually inputting the cost into the SIM dataset.

**Outlier Investigation**

Following this, it was noted that 3 patients exhibited cost data of more than twice that of the next largest medication costs (Figure 3). These three individuals were investigated further and following this; one individual was dropped because they had no prescription quantity data and as such it was entirely imputed; one individual had a prescription deleted because the quantity was so large that it was deemed in input error; and one individual was kept because there was no obvious reason for exclusion and therefore costs could be plausible.

**Figure 3:** Boxplot of medication costs per patient per year to show outliers which merited further investigation. Red line denotes twice the value of the next highest medication cost (€40,000).

**Confirmatory Analysis**

A confirmatory analysis was undertaken in which 50 patients per cohort had drug costs estimated as described above. All drug costs for these patients were also manually costed based on the string descriptors for their prescriptions and results compared. Coefficients from a univariate regression analysis, along with correlation coefficients, between the micro-costed drugs and the algorithm costed drugs, show a strong and statistically significant relationship (Table 2). This demonstrates that the algorithm is a strong predictor of a manual costing approach.

**Table 2:** Correlation coefficients (ρ) and regression coefficients between log of micro costs and log of algorithm costs.

| **Cohort** | **N** | **ln(Algorithm)** | **95% CI (Lo)** | **95%CI (Hi)** | **ρ** |
| --- | --- | --- | --- | --- | --- |
| **Non-asthma** | 50 | 0.96*** | 0.88 | 1.04 | 0.85 |
| **Mild Asthma** | 50 | 0.96*** | 0.90 | 1.02 | 0.92 |
| **SRA** | 50 | 1.02*** | 0.99 | 1.05 | 0.98 |
| **All** | 150 | 0.98*** | 0.95 | 1.01 | 0.97 |

*** Significant at the 99% level.

The difference in cost between methods (in log form) was compared against the average cost between methods (in log form), as further confirmatory analysis (3, 4) . This is to further investigate the level of agreement between the two methods, for example if the difference in patient costs between the two methods demonstrates a relationship with the average patient cost between the two methods, then this would imply a systematic bias in the algorithm costing. Furthermore if a large amount of observations (that is the difference in patient cost between the two methods) lie outside the 95% confidence intervals it would imply that the algorithm would be an inaccurate predictor of costs produced using a labour intensive approach.

**Figure 4:** Difference in logs between methods plotted against average of logs with repeated 95% outlier cuts.

Figure 4 shows that the algorithm approach is highly predictive of the manually based approach; the 95% confidence interval on the difference between the two approaches after exclusion of 11 or 21 outliers are small; around (36% above and 24% below excluding 11 out of 150 outliers - purple line in Figure 1; 16% above and 15% below excluding 21 outliers – red line).

**Healthcare Activity Data**

OPCRD sample (SIM) data contains information on each patient interaction with a health professional (referred to as activity). These activities are documented through the Read code system (5) in which descriptions of a patient activity with the GP are detailed in a hierarchy of description headings e.g. Read code ‘337..00’ relates to ‘Lung Function Testing’, Read codes beginning in ‘33’ relate to ‘Physiological function tests’ and Read codes beginning in ‘3’ relate to ‘Diagnostic procedures’.

**Consultation Ambiguity**

In order to calculate the total expenditure (TE) of all patients on all healthcare activity for the observable time period the quantity (the number of activities) and price (the cost of an activity) are needed. Knowing the type of consultation(s) (e.g. GP visit, Elective Inpatient Short visit, etc.) in relation to the activity, described through the Read code system, is crucial for assigning costs to the data. However we cannot be sure we will ascribe the correct cost to the interaction as the Read codes are ambiguous, for example a Read code on a specific date may refer to a cervical smear and this may have been carried out by a GP or a nurse for which costs would be different. To help ensure that the appropriate costs were assigned to activity, the following steps were taken:

1. Firstly, a list of all Read codes that occur in the OPCRD sample was extracted. Then a set of general rules used to assign consultation types to Read codes based on their description headings – e.g. an A&E consultation is assigned to all those codes that have a Read code heading of ‘Injury/Poisoning’. Full details of the general rules applied to this list are in Appendix 2.
2. Due to the ambiguity around Read code descriptions, there will be cases where the consultation type assigned to an interaction is inappropriate. For example, a GP or a nurse may carry out ‘diabetic monitoring’ (Read code ‘66A..00’). Two individuals examined the assignments from step 1, individually agreeing any changes in a manner akin to a systematic literature review. That is, they independently examined the assignments, shared their assignments including any changes to the original assignments and resolved any disagreements. Where uncertainty existed as to the correct Read code to be allocated after this exercise was complete, for example, a nurse or a GP could have performed the task concerned, both of these codes were documented and used for a High and Low scenario comparison.

**Monetising Consultation Data**

Unit costs for consultation types were obtained from the PSSRU (6). These costs were applied to each consultation type that was used in the Clinical data. Estimates in the PSSRU are more detailed for certain consultation types than others:

- For the least detailed, there was an average hourly cost of the consultation with no information on the duration of that consultation (blue in Table 3).

**Table 3:** Unit cost data coloured by information availability in the PSSRU 2013

- For some there were average face-to-face costs with upper and lower quartiles, where the duration of the consultation is implicit in the cost (green in Table 3).
- For some (e.g. GP and Nurse) there were hourly face-to-face costs with average times for each type (e.g. phone, surgery, home visit) made explicit (orange in Table 3).

For the estimates whereby average cost adjusted for the average duration of the consultation type, and upper and lower quartiles were specified (green), there was no requirement for adjustment and these estimates were applied directly to the clinical data.

For estimates where average hourly cost was specified but no information on the average duration of the consultation type was specified, it was assumed that these contacts lasted an hour, i.e. the average hourly cost was used. With the exception of GMS, which was to account clinicians administrative time. In this case research (7, 8) as well as personal comments found approximately 2.5 minutes was spent on patient administration. For the estimates (orange) and the estimates whereby average duration of contact was specified (blue), there was no information on upper and lower quartiles provided. In this instance, inter-quartile ranges were calculated using a standard gamma distribution with 10,000 simulated observations.

**Read Code Uniformity**

The structure of the data is such that a patient is observed every time they come in contact with a practitioner (e.g. an encounter with a GP) or the practitioner records information in relation to a patient (i.e. a GP records an administrative procedure, such as a letter being sent in relation to specific patient). These activities are denoted by a Read code (RC) per event date per patient.

In the patient data, where an event occurs (whether a patient encounter or related administration) there may be more than one entry for that date. For example a practitioner may record symptoms through the use of one RC, along with some diagnostic procedures (more RCs), followed by some administration (more RCs) after the patient may have left.

Difficulties arise in costing each event (as denoted by a single date) where multiple RCs occur on that date and the consultations allocated to each RC are not the same. For example, an RC relating to History and Symptoms may be assumed to relate to a GP consultation. However, if on the same date there was an RC relating to a fractured elbow, this additional RC raises the possibility that the History and Symptoms may more likely have been recorded by an A&E doctor and should be costed accordingly. The absence of another RC for that date (only history and symptoms) would lead us to conclude that it was more likely recorded by a GP and should be costed accordingly.

Below is a list of the rules applied to patient data in order to logically combine these RCs in a way that represents the most likely situation for each event while avoiding double counting.

***General Rules***

- Where only one RC was documented only one cost was assigned. For example, a GP office visit was assigned the cost of a GP office consultation. Where multiple Read codes at lower levels of the Read code hierarchy were associated with a single consultation – for example, where multiple tests or examinations might have been undertaken by a GP during one consultation on the same date – a single contact with associated cost was attributed on the assumption that all actions were part of one rather than multiple consultations on the same day. This would avoid double counting of consultations. Where more than one Read code was recorded on a particular date and they were not uniform – for example where an A&E visit and GP consultation are recorded with multiple activities being undertaken - the unit cost for the activity that was less frequently used was assigned. The latter approach was adopted based on the assumption that infrequent occurrences were more likely to be accurately recorded.

***Specific Rules***

The application of the general rules above covered the majority of consultations, although necessary as a start point for dealing with over 900,000 observations, will likely lead to inconsistencies. As such specific rules are outlined below in order to reduce the likelihood and magnitude of these inconsistencies.

- Where a GMS (administration) coded RC is recorded, this was added to the cost of the event, even if it occurs alongside other RCs. This is so that the administration carried out in relation to a patient, even if it occurs on the same date as the patient encounter, was not overlooked.
- Where a RC falls under the general description of ‘Laboratory Procedure’, an additional test cost was added to the event (9). Only one test cost was added per event regardless of how many laboratory procedures are documented on that date to avoid overestimating the cost of multiple tests on a single sample.
- Where ‘Seen in ….’, ‘Seen by ….’, ‘Discharged from….’ or ‘Letter from ….’ occurred, the consultation that is associated with this Read Code is added to the cost for that event. This shows that a patient has visited another practitioner elsewhere and their GP has recorded this event. Using just the general rules above, this event may be lost amongst other Read codes but actually documents an activity happening outside the patient’s interaction with their GP. So for example if ‘Seen in diabetes clinic’ occurs, the cost of an outpatient visit is added to the event date, regardless of what other Read codes are present on that event date. A cost of GP administration (GMS) is also added to each of these because it is assumed that the GP is handling this patient information. Additional code also ensured that if one of these occurred for a single event date then it was only costed once, this was based on a visual examination of the data whereby it was more likely that if one of the above occurred more than once for a single event date, it was most likely referring to the same referral rather than multiple referrals. Thus, not recording it in this way would more likely lead to overestimation of the true cost than underestimation without the rule.
- Where ‘vaccine’ or ‘vaccination’ occurred, the consultation was coded as a GP Nurse visit.
- Where a radiographer Read Code was observed this cost was added to the cost of the event, even if it occurs alongside other RCs.

**Results**

**Table 4:** List of service types along with their groupings (e.g. Hospital or GP related) for presentation of results (Figure 1 in paper).

| **Activity** | **Activity Code** | **Grouping** | **Scenario 1** | **Scenario 2** |
| --- | --- | --- | --- | --- |
| General Medical Service | GMS | GP | 18.62% | 17.76% |
| GP Surgery | GPS |  | 22.79% | 22.90% |
| GP Out of Hours | GPE |  | 1.87% | 1.99% |
| GP Telephone consultation | GPT |  | 5.53% | 5.55% |
| GP Nurse | NGP |  | 21.75% | 16.91% |
| Elective Inpatient | EI | Hospital | 0.41% | 6.01% |
| Non-Elective Inpatient (Long) | NEIL |  | 0.00% | 0.44% |
| Non-Elective Inpatient (Short) | NEIS |  | 2.91% | 0.00% |
| Specialist inpatient palliative care | SIPC |  | 0.01% | 0.01% |
| Day case | DC |  | 4.47% | 0.29% |
| Radiographer | RO |  | 7.96% | 7.98% |
| Accident and Emergency | A&E |  | 2.43% | 3.62% |
| Outpatient | O | Outpatient | 9.19% | 14.86% |
| Outpatient medical specialist palliative care attendance | SOPCM |  | 0.27% | 0.26% |
| Pharmacist | Pharm | Other Healthcare Providers | 0.16% | 0.00% |
| Optician | Opt |  | 0.49% | 0.00% |
| Dietitian | Diet |  | 0.12% | 0.00% |
| Mental Health Nurse | MHN |  | 0.05% | 0.05% |
| Community Nurse | CN |  | 0.15% | 0.14% |
| Community Nurse (Specialist) | CNS |  | 0.04% | 0.04% |
| Nurse Advanced | NA |  | 0.08% | 0.08% |
| Physiotherapist | Phy |  | 0.41% | 0.41% |
| Community Occupational Therapist | COT |  | 0.05% | 0.05% |
| Community Speech and Language Therapist | CSLT |  | 0.04% | 0.04% |
| Community Chiropodist/Podiatrist | CCP |  | 0.13% | 0.13% |
| Clinical Psychiatrist | Psy |  | 0.00% | 0.00% |
| Health Visitor | HV |  | 0.00% | 0.00% |
| Counselling Services | CS |  | 0.07% | 0.36% |

Proportion of occurrences for each consultation type in the data is also provided for each of the two scenarios, which were developed in the review stage where there was uncertainty as to the appropriate consultation to apply to Read codes.

**Table 5:** Mean cost per patient per year for clinical activity and drugs across all scenarios, as well as the average number of prescriptions per cohort (drug prescription count).

|  |  |  |  | **Cohort** | | |  |
| --- | --- | --- | --- | --- | --- | --- | --- |
|  | **Read Code Allocation Scenarios** | **PSSRU Consultation Estimate Bounds** |  | **Non-asthma (Rhinitis)** | **Mild Asthma** | **Severe Asthma** | **F-Test** |
| **Consultation Costs** | Low | Lower | Mean | 350 | 491 | 911 | <0.01 |
|  |  |  | S.Dev. | (546) | (630) | (907) |  |
|  |  | Middle | Mean | 445 | 625 | 1156 | <0.01 |
|  |  |  | S.Dev. | (733) | (848) | (1217) |  |
|  |  | Upper | Mean | 508 | 714 | 1320 | <0.01 |
|  |  |  | S.Dev. | (851) | (986) | (1410) |  |
|  | High | Lower | Mean | 743 | 1055 | 1880 | <0.01 |
|  |  |  | S.Dev. | (1489) | (1818) | (2329) |  |
|  |  | Middle | Mean | 962 | 1365 | 2426 | <0.01 |
|  |  |  | S.Dev. | (2018) | (2468) | (3156) |  |
|  |  | Upper | Mean | 1111 | 1579 | 2799 | <0.01 |
|  |  |  | S.Dev. | (2372) | (2902) | (3705) |  |
|  | | **BNF Allocation Estimator** |  | | | |  |
| **Therapy Costs** | | Median | Mean | 210 | 493 | 1734 | <0.01 |
|  |  |  | S.Dev. | (700) | (957) | (2369) |  |
|  |  | Mean | Mean | 212 | 487 | 1692 | <0.01 |
|  |  |  | S.Dev. | (790) | (947) | (2346) |  |
| **Therapy Count** | | | Mean | 25 | 44 | 118 | <0.01 |
|  |  |  | S.Dev. | (48) | (59) | (137) |  |

**Zero-inflated Poisson model**

The first stage regression analysis of non-asthma drugs identified that, when adjusting for confounders, patients with low exposure (mild asthma) would have an increase in the number of comorbidities of 0.4 (95% Confidence Interval (CI): 0.4 to 0.5), relative to no exposure patients (non-asthma). Patients with high exposure (SA) could expect a 1.3 (1.2 to 1.4) increase in the number of comorbidities relative to no exposure. Males had 0.3 (-0.4 to -0.25) fewer recorded comorbidities on average than females. Older age groups are associated with a statistically significantly greater number of additional comorbidities

**Table 6**: Average marginal effects from zero-inflated poisson regression

| Cohort (Base: Non-asthma) | Additional Comorbidities | Lower CI (95%) | Upper CI (95%) |
| --- | --- | --- | --- |
| Mild asthma | 0.43*** | 0.356 | 0.502 |
| Severe asthma | 1.29*** | 1.155 | 1.416 |
| Sex (Base: Female) |  |  |  |
| Male | -0.31*** | -0.38 | -0.245 |
| Age Group (Base: <46 yrs) |  |  |  |
| 46 - 60 | 0.59*** | 0.513 | 0.664 |
| 61 - 70 | 1.19*** | 1.102 | 1.286 |
| > 70 | 1.88*** | 1.785 | 1.982 |
| Region (Base: London) |  |  |  |
| South | -0.14* | -0.292 | 0.004 |
| East | -0.09 | -0.232 | 0.05 |
| Midlands | -0.05 | -0.179 | 0.08 |
| North | 0.15** | 0.009 | 0.283 |
| Missing and excluding England | -0.01 | -0.164 | 0.138 |

**Appendices**

**Appendix 1: Read codes and BNF categories for identifying asthma**

**Table 7:** Read codes and terms

| **Read code** | **Read term** |
| --- | --- |
| H33..00 | Asthma |
| H33..11 | Bronchial asthma |
| H330.00 | Extrinsic (atopic) asthma |
| H330.11 | Allergic asthma |
| H330.12 | Childhood asthma |
| H330.13 | Hay fever with asthma |
| H330.14 | Pollen asthma |
| H330000 | Extrinsic asthma - no status |
| H330011 | Hay fever with asthma |
| H330100 | Extrinsic asthma + status |
| H330111 | Extrinsic asthma + attack |
| H330z00 | Extrinsic asthma NOS |
| H331.00 | Intrinsic asthma |
| H331.11 | Late onset asthma |
| H331000 | Intrinsic asthma - no status |
| H331100 | Intrinsic asthma + status |
| H331111 | Intrinsic asthma + attack |
| H331z00 | Intrinsic asthma NOS |
| H332.00 | Mixed asthma |
| H333.00 | Acute exacerbation of asthma |
| H33z.00 | Asthma unspecified |
| H33z000 | Status asthmaticus NOS |
| H33z011 | Severe asthma attack |
| H33z200 | Late-onset asthma |
| H33zz00 | Asthma NOS |
| H33zz11 | Exercise induced asthma |
| H33zz12 | Allergic asthma NEC |
| H33zz13 | Allergic bronchitis NEC |

**Table 8:** BNF categories for asthma and corticosteroids

| **BNF category** |
| --- |
| Compound bronchodilator preparations |
| Corticosteroids (inhaled for respiratory conditions) |
| Leukotriene receptor antagonists |
| Selective beta-2-agonists |
| Theophylline |
| Glucocorticoid therapy |

**Appendix 2: Quantity cut-off points for therapy string descriptions**

Cut-off = 8

String list: inh INH haler HALER

Cut-off = 7

String list: oint tab TAB cap CAP grans stick lancet loz mix

Cut-off = 5

String list: crm liq drp drop DROP paste gel GEL syrp spray soln supp pwdr

Cut-off = 2

String list: dres strip inj vaccine susp patch application Gluta Fresu Proceli Lifestyle Barkat Wellfoods Ensure Forti Livwell Juvela Ener Schar Maxijul Enlive

**Appendix 3: General allocation of consultation types to Read codes**

Top level Read code hierarchies (Table 8 below) are investigated across each Read code term in the data and assigned consultation types at this level. Further investigation is then carried out to identify and document alternative consultation types based on lower level Read code hierarchies. It should be noted that assigning consultation types to Read Codes involves uncertainty and assigning these consultation types to patient interactions will require using information on the Read Code combinations per interaction (e.g. ‘History and Symptoms’ are coded as a GP visit but if accompanied with an operation then may be coded as part of an Inpatient Episode).

#### **0 – Occupations**

Coded as GP General Medical Service unless alternative information exists alongside specific patient activity (recorded as a single date in the data), e.g. operation or procedure.

#### **1 – History/symptoms**

Coded as GP surgery (GPS) visit unless alternative information exists alongside specific patient activity (recorded as a single date in the data), e.g. operation or procedure. ‘Read Code Administration’ is coded as a General Medical Service consultation.

#### **2 – Examination/Signs**

Coded as GP surgery visit unless alternative information exists alongside specific patient activity (recorded as a single date in the data), e.g. operation or procedure.

#### **3 – Diagnostic Procedures**

Coded as an Outpatient hospital visit.

#### **4 – Laboratory Procedures**

Coded as GP surgery visit unless alternative information exists alongside specific patient activity (recorded as a single date in the data), e.g. operation or procedure.

#### **5 – Radiology/physics in medicine**

Coded as a Radiographer hospital visit.

#### **6 – Preventive Procedures**

Coded as a GP surgery visit. Exceptions:

##### **62 & 63 – Patient pregnant & Birth details**

Low cost - Nurse GP visit

##### **67 – Counselling/health education**

Coded as a Nurse GP visit

#### **7 – Operations, procedures, sites**

Coded as an Elective Inpatient visit. Ambulatory observation coded as A&E and Vaccination coded as GPS.

#### **8 – Other therapeutic procedures**

Coded as a GP surgery visit, unless telephone is mentioned then coded as GP telephone call.

#### **9 – Administration**

Coded as GP General Medical Service. Where ‘Attends….’, ‘Attending….’, ‘seen in….’ or ‘seen by….’ occurs then this is coded as an outpatient visit, except for where specific consultation is mentioned. Coded as GP telephone call where ‘telephone’ is mentioned. Coded as an out of GP surgery visit where ‘home visit’ is mentioned.

##### **987 & 9kc – ‘FP/MS – minor surgery claim’ & ‘Minor surgery – enhanced services administration’**

Coded as a GP surgery visit

#### **A – Infectious and parasitic diseases**

Coded as a GP surgery visit.

#### **B – Neoplasms**

Coded as a GP surgery visit.

#### **C – Endocrine, nutritional, metabolic and immunity disorders**

Coded as a GP surgery visit.

#### **D – Diseases of blood and blood-forming organs**

Coded as GP surgery visit.

#### **E – Mental Disorders**

Coded as a visit to a Community Mental Health Nurse visit. Where there is ambiguity as to relation to Mental Disorders (e.g. Isolated tenderness of patella, Read Code: EMI), then coded as a General Practice Surgery (GPS) visit. Uncertainty as to appropriate alternative to Mental Health Nurse thus GP Surgery used for consistency.

#### **F – Nervous system and sense organ diseases**

Coded as a GP surgery visit.

#### **G – Circulatory system disorders**

Coded as a GP surgery visit. Exceptions:

##### **G57 & G58 – Cardiac dysrhythmias & Heart failure**

Coded as an Accident and Emergency visit.

#### **H – Respiratory system diseases**

Coded as a GP surgery visit.

#### **J – Digestive system diseases**

Coded as a GP surgery visit.

#### **K – Genitourinary system diseases**

Coded as a GP surgery visit.

#### **L – Complications of pregnancy, childbirth and the puerperium**

Elective Inpatient (maybe vice versa, depending on Midwife costs)

#### **M – Skin and subcutaneous tissue diseases**

Coded as a GP surgery visit.

#### **N – Musculoskeletal and connective tissue diseases**

Coded as a GP surgery visit.

#### **O – Undetermined**

Coded as a GP surgery visit.

#### **P – Congenital anomalies**

Coded as a GP surgery visit.

#### **Q – Perinatal anomalies**

Elective Inpatient

#### **R – [D]Symptoms, signs and ill-defined conditions**

Coded as a GP surgery visit.

#### **S – Injury and poisoning**

Coded as an Accident and Emergency visit. Where ‘operation complication’ is mentioned then coded as a Non-Elective Inpatient Short (NEIS) visit.

#### **T – Causes of injury and poisoning**

Coded as an Accident and Emergency visit.

#### **U – [X] External causes of morbidity and mortality**

Coded as an Accident and Emergency visit.

#### **X – Undetermined**

Coded as an GP Surgery visit.

#### **Y – Undetermined**

Coded as an Accident and Emergency visit.

#### **Z – Unspecified conditions**

Coded as an Outpatient hospital visit. Where ‘referral to’ or ‘refer to’ is mentioned then coded as a GP Surgery visit, where ‘Accident and Emergency’ is mentioned then coded as an A&E visit.

##### **Z1 – Nursing care**

Coded as a GP Nurse visit.

**Table 9:** Highest level categories of the Read code system

**References**

1. Prescription Cost Analysis at Northern Ireland. In: HSC Business Services Organisation, editor. 2013 ed. <http://www.hscbusiness.hscni.net/services/2523.htm2013>.

2. Joint Formulary Committee, Royal Pharmaceutical Society of Great Britain. British national formulary: Pharmaceutical Press; 2013.

3. Bland MJ, Altman DG. Statistical methods for assessing agreement between two methods of clinical measurment. The Lancet. 1986;327(8476):307-10.

4. Bland JM, Altman DG. Statistical methods for assessing agreement between two methods of clinical measurement. International Journal of Nursing Studies. 2010;47(8):931-6.

5. Health and Social Care Information Centre. Read codes 1985 [Available from: <http://systems.hscic.gov.uk/data/uktc/readcodes>.

6. PSSRU. Unit Costs of Health and Social Care 2013. Canterbury, England: University of Kent; 2013.

7. Chen M, Hollenberg J, Michelen W, Peterson J, Casalino L. Patient Care Outside of Office Visits: A Primary Care Physician Time Study. J GEN INTERN MED. 2011;26(1):58-63.

8. Gottschalk A, Flocke SA. Time Spent in Face-to-Face Patient Care and Work Outside the Examination Room. Annals of Family Medicine. 2005;3(6):488-93.

9. Information Services Division S. Laboratory Services - Costs 2015 [Available from: <http://www.isdscotland.org/Health-Topics/Finance/Costs/Detailed-Tables/Laboratory.asp>.
